# Paragangliomas of the head and neck: clinical, morphological and immunohistochemical aspects

**DOI:** 10.1590/S1516-31802001000300006

**Published:** 2001-05-02

**Authors:** Pedro de Alcântara de Andrade, Abrão Rapoport, Venâncio Avancini Ferreira Alves, Odilon Victor Porto Denardin, Josias de Andrade Sobrinho, Marcos Brasilino de Carvalho

**Keywords:** Paragangliomas, Markers, AgKi67, Chromogranin, S100 protein, Paragangliomas, Marcadores, AgKi67, Cromogranina e proteína S100

## Abstract

**CONTEXT:**

Protein marker positivity can assist in the definition of the therapeutic approach towards head and neck paragangliomas. The establishment of the therapeutic approach should incorporate the results of such an investigation.

**OBJECTIVE:**

To establish criteria for benignancy and malignancy of vagal and jugular-tympanic paragangliomas, via the study of the relationships of sex, age, tumor size, duration of complaints, site, family history, presence of metastases, treatment, histological architecture and cell type with the immunohistochemical reactions to S100 protein, chromogranin and AgKi67.

**DESIGN:**

A retrospective study of histological and clinical records.

**SETTING:**

The Heliópolis and Oswaldo Cruz tertiary general hospitals, São Paulo.

**SAMPLE:**

8 cases of head and neck paragangliomas.

**MAIN MEASUREMENTS:**

Determination of degree of positivity to paragangliomas via immunohisto-chemical reactions.

**RESULTS:**

1). The protein markers for the principal cells (AgKi67 and chromogranin) were sensitive in 100% of the tumors when used together. 2). S100 protein was well identified in the cytoplasm and nucleus of sustentacular cells and underwent reduction in the neoplasias.

**CONCLUSIONS:**

Chromogranin was proven to be a generic marker for neuroendocrine tumors; S100 protein was positive in all 8 cases and the AgKi67 had low positivity in all cases.

## RESUMO

**CONTEXTO:**

. A marcação positiva pode auxiliar na escolha da terapêutica dos paragangliomas de cabeça e pescoço. A definição do tratamento deve ser realizada incorporando o resultado desses testes.

**OBJETIVO:**

Por meio do estudo da relação do sexo, idade, tamanho de lesão, tempo de queixa, localização, história familiar, metástase, tratamento, arquitetura, tipo celular com as reações imunohistoquímicas para proteína S100, cromogranina e AgKi67, estabelecimento de critérios de benignidade e malignidade dos paragangliomas vagais e júgulo-timpânicos.

**TIPO DE ESTUDO:**

Estudo retrospectivo de arquivos histológicos e clínicos.

**LOCAL:**

Hospital Heliópolis e Oswaldo Cruz, São Paulo.

**PARTICIPANTES:**

Oito casos de paragangliomas de cabeça e pescoço.

**VARIÁVEIS ESTUDADAS:**

Grau de positividade para paragangliomas com reação imunohistoquímica.

**RESULTADOS:**

Os marcadores protéicos para as células principais (AgKi67 e cromogranina) foram sensíveis em 100% das neoplasias quando usados conjuntamente; A proteína S100 foi bem identificada no citoplasma e núcleo das células sustentaculares e sofre diminuição nas neoplasias.

**CONCLUSÕES:**

a cromogranina comprovou ser um marcador genérico para os tumores neuroendócrinas, a proteína S100 foi positiva nos oito casos e o Ki67 foi baixo em todos os casos.

## INTRODUCTION

Head and neck paragangliomas are rare and are true neuroendocrine neoplasias. They originate from various paraganglia,^1^ members of a complex and intriguing family of endocrine cells that present centripetal distribution with a tendency towards symmetry, extending from the middle ear and skull base region to the pelvic floor.^2^

All these cells are derived from the neural crest and form the diffuse dispersed neuroendocrine system. These kinds of cells are found in the central and peripheral nervous system and in many classic endocrine organs, for example the adrenal gland. They are distributed in the majority of body tissues, organized as isolated cells or in groups of 3 or 4 cells, exhibiting a heterogeneity of phenotypes that makes for structural diversity. There are basically two kinds of cells: the principal cells and the sustentacular cells. The former are rounded, with a dark central nucleus, eccentric, with eosinophilic granules in their cytoplasm, identified as neurosecretory granules by means of histochemical or immunohisto-chemical reactions, or using electron microscopy, with a halo around the membrane rim also being noted. They are grouped in nests and can be classified as light, dark and pyknocytic. The sustentacular cells, located on the periphery of these nests of principal cells (Zellballen model) are pale, with elongated nuclei and indistinct cytoplasm. Near to the principal cells, axonal processes known as the mesaxonia can be observed. Endothelial and pericytic cells, and occasionally mast cells, are also found in paraganglia.^3^

Head and neck paraganglia have an intimate relationship with vascular and neural structures.^4,5^ They have a strategic localization that allows the development of a chemoreceptor function in response to alterations in gas concentrations in arterial blood.^6^ Another important aspect is the chemoreceptor-sensitive function of the carotid bodies, which are sensitive to p0_2_, pH and pCO_2_ alterations in the blood, in which the chronic hypoxemia stimulates the carotid body hyperplasia.^7^

These paragangliomas present approximately the same architecture as a normal paraganglion, with some variation in size and shape, but the main difference in this neoplasia is the proliferation of principal cells in nests surrounded by sustentacular cells that represent 1 to 5 % of the cells of a paraganglioma and form a prominent vascular network.^8^ Three architectural patterns have been described:^9^ 1) the normal or Zellballen model; 2) angiomatous, with large spindle or crescent-shaped principal cells and the appearance of capillaries; and 3) adenomatous, with a marked similarity between the principal cells and the epithelial cells, i.e. polyhedral cells with abundant cytoplasm and columnar arrangement.

Carotid body paragangliomas are different from hyperplasia in that they present proliferation of the principal cells whereas in hyperplasia there is proliferation of the principal and sustentacular cells.^10^ The majority of head and neck paragangliomas contain neurosecretory granules with vasoactive substances like epinephrine, norepinephrine, dopamine and serotonin.^11^ The fact that these catecholamines are present does not signify that there is a biological effect.^12^ Nevertheless, the measurement of vanil-mandelic acid is recommended as a screening method when these tumors are suspected.^13,14^

The clinical presentation is usually asymptomatic. Pain, hoarseness, dysphagia, Horner's syndrome, tinnitus and hearing loss may occasionally be presented. There is evidence that these paragangliomas present as family histories,^15, 16^ which has led to studies of oncogene identification.^17,18^ Most paragangliomas are benign, but 6 to 9% of head and neck paragangliomas present histologically and biologically malignant behavior,^19^ with the appearance of mitotic cells, cell pleomorphism and central necrosis in Zellballen areas). Malignancy, widely discussed by many authors, is only fully characterized by distant metastasis,^20^ with there being concern regarding the lack of identified histopathological factors (i.e. the cell atypia, nuclear polymorphism and local invasion factors that are typical of malignancy).^21^ Histological comparison between benign and malignant paragangliomas does not demonstrate any differences,^22^ and the most important factor among these histological factors seems to be the site of the tumor itself. The incidence of metastasis in para-aortic paragangliomas ranges from 28 to 42%. The knowledge available at present still does not allow the reasons for this difference in relation to head and neck paragangliomas to be explained.^21^

Using electron microscopy, a pattern of diminution or absence of sustentacular cells was found in the tumor architecture of malignant paragangliomas.^10^ This was the first histological observation step towards predicting the clinical behavior of such tumors. It was followed by the introduction of protein markers using immunohistochemical methods, in order to identify prognostic factors in head and neck paragangliomas.

**Figure 1:**
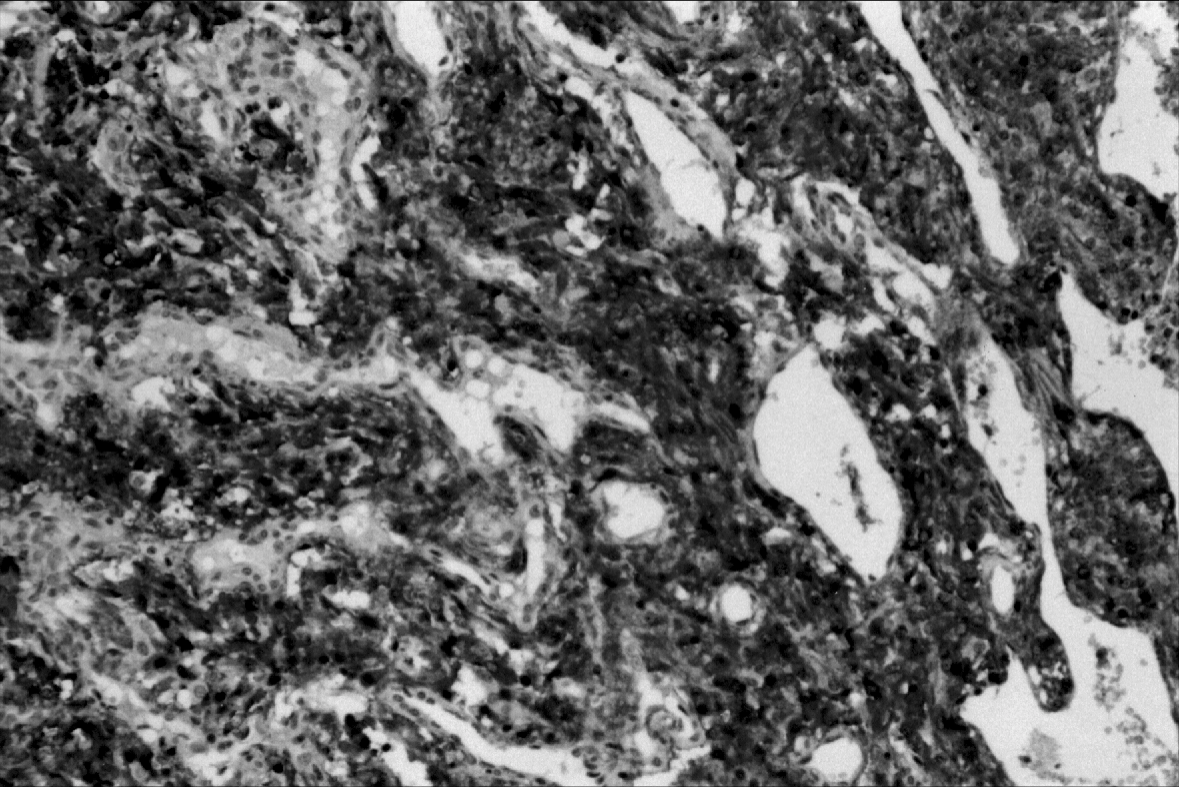
Cells stained by Cromogranin.

**Figure 2:**
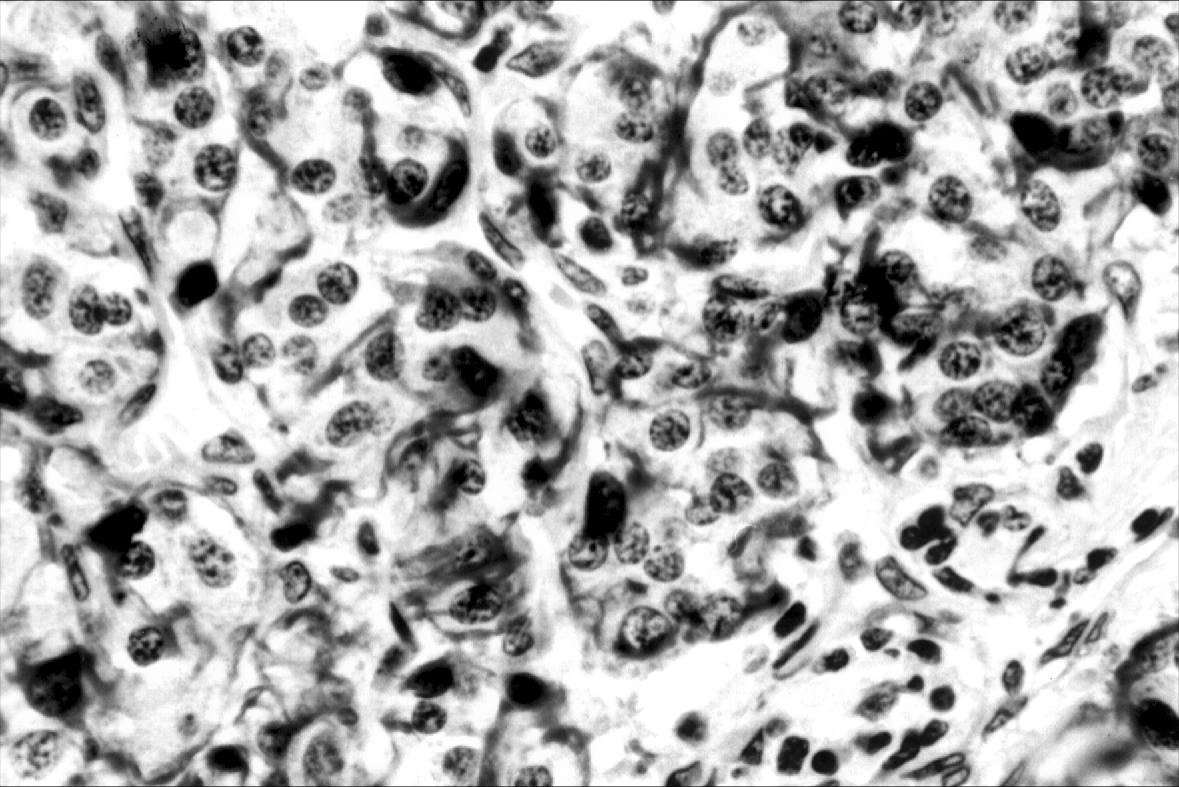
Sustentocullar cells stained by S100 protein.

**Figure 3:**
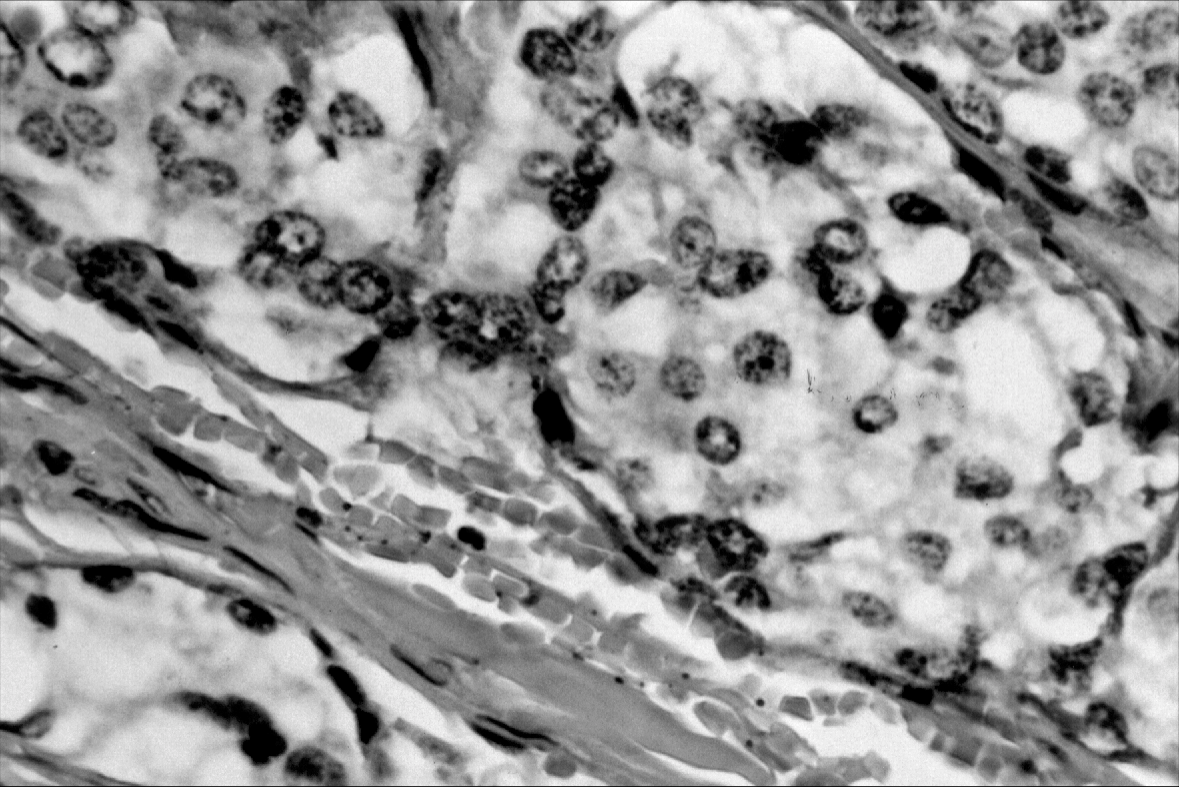
AgKi67 staining two nucleous.

Various tumor markers have been tested. Some have confirmed the neuroendocrine origin of the neoplasia and others have shown up strong suspicions of malignant behavior.^4^ In these latter cases, even in the absence of distant metastases, there was greater tumor aggressiveness, either with the invasion of adjacent structures or with disease recurrence. In the present study, three immunohisto-chemical markers were used: chromogranin (Figure 1), S100 protein (Figure 2) and AgKi67 (Figure 3).

Chromogranin, the main marker for neuroendocrine cells, is a structural protein found in neurosecretory granules of principal cells. Its function is to stabilize the intra-cellular matrix of neurosecretory granules, thereby showing itself to be an excellent indicator of neuroendocrine differentiation. Well-differentiated tumors contain more neurosecretory granules and the undifferentiated ones have less of them.^4^

S100 protein, a dimeric 21-Kd protein bonding with calcium, was isolated from the nervous system. It has been identified in sustentacular cells of autonomous ganglia, adrenal medullas and extra-adrenal paraganglia. It may also be identified in sustentacular cells of neural and neuroendocrine neoplasias. It is localized in the cytoplasm and nuclei of the sustentacular cells of extra-adrenal paraganglia. In extra-adrenal paraganglioma, it has been demonstrated that all the benign tumors contain sustentacular cells, whereas these are seen to be absent in malignant tumors.^4^

The nuclear antigen AgKi67 (MiB-1) is a protein in all phases of the cell cycle except the G0 phase, and has a direct relationship with the growth rate of a cell population. It is an excellent marker for cell proliferation.^23^

The question thus arises of whether immunohistochemistry could be helpful in the identification of benign paragangliomas and their behavior.

The objective of this work was therefore, in summary, to establish clinical, histological and immunohistochemical criteria for benignancy and malignancy, by means of the retrospective analysis of 8 cases of carotid body and jugular-tympanic paragangliomas.

## METHODS

From an analysis of the records of the Head and Neck Service at Heliópolis Hospital, 8 cases of head and neck paragangliomas were found to have been submitted to surgical procedure at the Head and Neck Service between 1977 and 1995. The following data were analyzed: time of complaint, family history, sex, age, site of the tumor, its size (considering sizes as greater or less than 5 cm) and metastases.

For the present study, new histological sections of thickness 3 micrometers were obtained from the old paraffin blocks. The slides were stained with hematoxylin and eosin and other slides were also prepared for the immunohistochemical reactions. All slides were reviewed by the same pathologist. The architecture of the paraganglioma was evaluated and classified as follows:

- N = nest pattern (classic or Zellballen pattern), with sustentacular cells (elongated cells with indistinct cytoplasm) surrounding groups of principal cells (rounded or polyhedral cells that are eccentric, with granular cytoplasm) and vascular stromata;
- A = Adenomatous: principal cells with marked similarity to epithelial cells, i.e. polyhedral cells with abundant cytoplasm organized in columns, surrounded by sustentacular cells and vascular stromata;
- V = angiomatous: spindle-shaped principal cells, similar to endothelial cells, surrounded by sustentacular cells and vascular stromata;
- S = solid: basically consisting of various polymorphic principal cells, with few vascular stromata.

The predominant cell type in paragangliomas is the principal cell, accounting for 50 to 90% of the total number of cells. These cells are rounded, large, with prominent nuclei, eosinophilic cytoplasm rich in granules and ranging from 2 to 10 micrometers in diameter. These cells may vary in size and shape and were classified as follows: a) C = principal cells: 2 to 10 micrometers in diameter; b) D = small cells: less than 2 micrometers in diameter; c) E = giant cells: more than 10 micrometers in diameter; d) F = fusiform cells: spindle-shaped cells, similar to vascular endothelium.

Cell atypia were evaluated and defined in terms of variation in shape and cell volume, the increase in the nucleus-cytoplasm relationship, irregularities in nuclear rims, nuclear hyperchromatism and the presence of nucleoli. They were semi-quantified as 0 (absent), 1 (mild), 2 (moderate) and 3 (accentuated).

In the immunohistochemical methods, three protein markers were evaluated: AgKi67, chromogranin and S100 protein with amplification using the streptavidin-biotin-peroxidase complex. The immunohistochemical reactivity for AgKi67 (AcMiB-1) was evaluated by quantitative analysis: a) 1 = rare cells (less than one per field at 400x magnification); b) 2 = moderate number of positive cells (up to 25% of cells); c) 3 = high proportion of positive cells (greater than 25%).

As it was already known that there would not be any neoplasias unaccompanied by cell proliferation, then if there were any case with a negative reaction to chromogranin, this would not be considered valid and would therefore act as a further control for the reaction. The immunohistochemical reactivity to chromogranin (for principal cells) and S100 protein (for principal and sustentacular cells) was evaluated by the following semi-quantification: a) 0 = negative; b) 1 = rare stained cells (less than 1 per field at 400x magnification); c) 2 = moderate positivity (up to 25% of the cells); d) 3 = numerous positive cells (more than 25% of the cells).

## RESULTS

The distribution according to sex was 7 (87.5%) of women and 1 (12.5%) man, with a mean age of 39.5 years (range 23 – 77 years). At the time of the complaint, six patients (75%) reported that the time of onset of the lesion had been more than 1 year earlier and 2 patients (25%) reported it as being less than 1 year earlier. The main complaint in 7 cases (87.5%) was a painless cervical tumor and in 1 case (12.5%) it was hearing loss and ear pain.

The distribution of patients in relation to the size of the tumor showed that 4 of them had a lesion of more than 5 cm and 4 less than 5 cm. The follow-up ranged from 1 to 132 months and no case of distant metastasis was noted.

The main histopathological and immunohistochemical findings are shown in Table 1 and Figures 1, 2, and 3.

**Table 1:** Distribution of patients and histopathological findings

CASE	Architecture	Cell type	Atypia	AgKi67	S100 sustentacular/principal cell	Chromogranin
1	N	C	0	1	1/0	1
2	N+S	C	1	1	3/2	3
3	N+V	C	3	1	3/2	3
4	S+N	C	2	1	3/1	1
5	N+S	C	2	1	2/0	2
6	N	C	2	1	3/2	2
7	S	C	2	1	1/0	2
8	V	F	1	2	2/0	3

*N = nest pattern; S = solid pattern; V = angiomatous, C = principal cells; F = spindle cells.*

There was a predominance of the nest pattern (5 cases: nos. 1, 2, 3, 5 and 6) and in 3 of them this was associated with other patterns (cases 2 and 5 with solid patterns, and case 3 with an angiomatous pattern). The predominant cell type was principal cells (7 cases: nos. 1, 2, 3, 4, 5, 6 and 7) and the remaining case had spindle-shaped cells as the main type (case 8).

Table 2 shows the relationships between tumor size and cell atypia, chromogranin reactivity, AgKi67 reactivity and positivity to S100 protein reaction for sustentacular cells.

**Table 2:** Relation between tumor size and cell atypia, chromogranin and AgKi67 reactivity, and sustentacular cell positivity to S100 protein reaction.

	Tumor (size)	Atypia	Chromogranin	AgKi67	S100 protein	
	-/+	2 (25%)	1(12.5%)	3(37.5%)	0	
< 5						
	++/+++	2 (25%)	3(37.5%)	1(12.5%)	4	(50%)
	-/+	1 (12.5%)	1(12.5%)	4(50%)	2	(25%)
> 5						
	++/+++	3 (37.5%)	3(37.5%)	0	2	(25%)

*- = negative; + = rare staining cells; ++ = moderate positivity; +++ = high positivity.*

The relationships between cell proliferation among principal cells and chromogranin, and between sustentacular cells and S100 protein, are shown in Table 3.

**Table 3:** Principal cell positivity to chromogranin and sustentacular cell reactivity to S100 protein

Chromogranin (principal cells)	S100 (sustentacular cells)	
	-/+	++/+++
-/+	1 (12.5%)	1 (12.5%)
++/+++	1 (12.5%)	5 (62.5%)

*- =negative; + =rare staining cells; ++ =moderate positivity; +++=high positivity.*

The clinical-pathological characteristics are presented in Table 4, in which it can be noted that 5 cases (62.5%) had a moderate to numerous quantity of cells reactive to S100 and chromogranin (cases 2, 3, 5, 6 and 8).

**Table 4:** Clinical-pathological aspects of the sample

Case no.	age/sex	place	symptom	histology	metastasis	Treatment	follow-up
1	23/F	carotid	12 months	N	-	Surgery	6 months
2	77/F	carotid	6 months	N+S	-	Surgery	13 months
3	30/M	carotid	6 months	N+V	-	Surgery	132 months
4	32/F	carotid	48 months	S+N	-	Surgery	1 month
5	47/F	carotid	12 months	N+S	-	Surgery	89 months
6	32/F	carotid	20 months	N	-	Surgery	1 month
7	36/F	carotid	12 months	S	-	Surgery	12 months
8	39/F	tympanic	24 months	V	-	Surgery	5 months

*Classic Pattern = Zellballen; V = angiomatous; S = solid.*

## DISCUSSION

The uncertain nature of head and neck paragangliomas makes their therapeutic management controversial. The clinical and epidemiological understanding still allows for some liberty of choice that discourages standardization of the approach to this disease. It is not clear why certain paragangliomas with same histopathological pattern follow different courses. These continuing uncertainties have justified new research ranging from epidemio-logical to cytogenetic studies, with the aim of identifying the biological behavior of the tumor.

The symptoms of these tumors are volume-dependent, such that their slow growth leads to the characteristics of an insidious disease. In a study among inhabitants of higher altitudes,^24^ it was found that the tumors evolved more rapidly. In that study, the female to male ratio was 8.3:1 with a mean age of 49 years. Tumors of the carotid body represented 79% of the cases of tumors of the para-pharyngeal space and the incidence of malignancy was 3.3%. The size of the tumor ranged from 2 to 12 cm (mean of 5.4 cm) and 1% of the cases had a family history.^24^ Such results have been confirmed by other authors.^19^

In the present study, the mean evolution time for the disease was 17.5 months, with women predominating (7:1) and a mean age of 39.5 years old. Most of the tumors (87.5%) were from the carotid body and 12.5% from the tympanic cavity. The mean size of the tumor was 4.81 cm and no reports of a family history were found. The female predominance differs from reports in the literature, except those from high altitudes. The mean age, size of tumor and site distribution were similar to those reported in the literature. There were no cases of multicentricity, family history, relapse and malignancy. The low number of cases could have led to a bias.

Cell atypia were found in cases 2, 3, 4, 5, 6, 7 and 8, of which five (cases 3, 4, 5, 6 and 7) had moderate to accentuated atypia. In addition, cellular necrosis was found in cases 3 and 5. The cell atypia data were correlated to the size of the tumor and this size was related to the reaction to AgKi67, chromogranin and S100 protein. In tumors bigger than 5 cm, 37.5% presented moderate to accentuated atypia. The relationship between size of tumor and tumor markers was not significant.

In studies using optical and electron microscopy,^10^ an absence of sustentacular cells has been noted in malignant paragangliomas^24^ and their metastases. This has triggered a new era in the study of paragangliomas. Sustentacular cells, previously less easily identifiable under the optical microscope because of confusion with vascular pericytes, have become better understood using electron microscopy.

Nevertheless, the task of identification has remained difficult and slow. We have simplified the task via immunohistochemical techniques, enhancing diagnoses and providing correlations with other aspects that assist in understanding the biological behavior of these tumors.

In a study using protein markers,^4^ two excellent markers for principal cells (of which there are greater quantities than of sustentacular cells) were detected that, when used in combination, gave 100% sensitivity for neuroendocrine neoplasms. These markers are divided into two groups: (1) enzymatic markers, represented by AgKi67, and (2) specific protein granules, represented by chromogranin. Their reactions can differentiate the true cases of paragangliomas from carcinomatous tumors. Chromogranin and AgKi67 correlate with the cell differentiation patterns of these tumors. Tumors that are more differentiated are more similar to normal tissue and, in consequence, they contain more cytoplasmic granules and react more intensely to these markers. On the other hand, undifferentiated tumors have less reactivity to chromogranin and AgKi67. In that same study,^4^ an absence of S100 protein was noted in malignant paragangliomas. Some studies have found different degrees of reactivity to these markers for principal cells, with the loss of reactivity being related to malignancy.^25^ Controversy persists regarding the real decrease in sustentacular cells in malignant tumors. One important point is that there is no case of malignant paraganglioma with sustentacular cells.^24^

S100 protein is well identified in the cytoplasm and nuclei of sustentacular cells and undergoes diminution in neoplasias. The presence of numerous sustentacular cells is highly associated with benign paragangliomas. The inverse is also true: a lack of sustentacular cells is associated with more undifferentiated and therefore malignant tumors. This consumption or disappearance of sustentacular cells still does not have any explanation.

Comparing the results of the present study, the immunohistochemical reactions were unspecific and variable. On the other hand, in the relationship between chromogranin in principal cells and S100 in sustentacular cells, 62.5% of the reactions were moderate to accentuated. These data may represent the course of the cases of benign disease, i.e. an absence of recurrence and metastasis. This affects the treatment because, depending on the site of tumor, its size and the patient's age, a more aggressive adjuvant treatment may be avoidable. If this information were available before treatment, we would certainly be more conservative and might even contra-indicate elective surgical treatment.

Radiotherapy may be an alternative treatment. In one study,^26^ fibrosis was observed between sustentacular cells and the perivascular network in irradiated patients. There was control of tumor growth despite the lack of noticeable effect on principal cells.^26^ In patients over 60 years old and at high risk in relation to anesthesia, this may be the treatment of choice. Such a choice is based exclusively on clinical experience and can be justified by the insidious behavior of the disease.^27^

We believe that present-day advances in biochemical science allow a much more solid therapeutic approach guided by prognostic factors. Faced with a case of head and neck paraganglioma, in addition to the detailed clinical analysis, imaging studies and anatomo-pathological studies of the surgical specimen, we can make use of immunohistochemical methods in assisting us to differentiate between benign and malignant tumor cases.

## CONCLUSION

We can confirm that chromogranin proved to be a generic marker for neuroendocrine tumors, the S100 protein was positive in all cases and the cell proliferation marker AgKi67 showed low positivity in all cases but one. Finally, 62.5% of the cases showed moderate to strong reaction to chromogranin and S100 protein, which was consistent with the benign evolution of these tumors.
